# Case report: Avoiding intolerance to antipsychotics through a personalized treatment approach based on pharmacogenetics

**DOI:** 10.3389/fpsyt.2024.1363051

**Published:** 2024-03-19

**Authors:** Liam Korošec Hudnik, Tanja Blagus, Sara Redenšek Trampuž, Vita Dolžan, Jurij Bon, Milica Pjevac

**Affiliations:** ^1^ Centre for Clinical Psychiatry, University Psychiatric Clinic Ljubljana, Ljubljana, Slovenia; ^2^ Pharmacogenetics Laboratory, Institute of Biochemistry and Molecular Genetics, Faculty of Medicine, University of Ljubljana, Ljubljana, Slovenia; ^3^ Department of Psychiatry, Faculty of Medicine, University of Ljubljana, Ljubljana, Slovenia

**Keywords:** gene-drug interaction, psychopharmacology, extrapyramidal adverse effects, personalized treatment, antipsychotics

## Abstract

**Introduction:**

The standard approach to treatment in psychiatry is known as “treatment as usual” (TAU), in which the same types of treatment are administered to a group of patients. TAU often requires numerous dose adjustments and medication changes due to ineffectiveness and/or the occurrence of adverse drug reactions (ADRs). This process is not only time-consuming but also costly. Antipsychotic medications are commonly used to treat various psychiatric disorders such as schizophrenia and mood disorders. Some of the inter-individual differences in efficacy and ADRs observed in psychopharmacotherapy can be explained by genetic variability in the pharmacokinetics and pharmacodynamics of antipsychotics. A better understanding of (in)efficacy and possible ADRs can be achieved by pharmacogenetic analysis of genes involved in the metabolism of antipsychotics. Most psychotropic drugs are metabolized by genetically variable CYP2D6, CYP1A2, CYP3A4, and CYP2C19 enzymes. To demonstrate the utility of pharmacogenetic testing for tailoring antipsychotic treatment, in this paper, we present the case of a patient in whom a pharmacogenetic approach remarkably altered an otherwise intolerant or ineffective conventional TAU with antipsychotics.

**Methods:**

In this case report, we present a 60-year-old patient with psychotic symptoms who suffered from severe extrapyramidal symptoms and a malignant neuroleptic syndrome during treatment with risperidone, fluphenazine, aripiprazole, brexpiprazole, and olanzapine. Therefore, we performed a pharmacogenetic analysis by genotyping common functional variants in genes involved in the pharmacokinetic pathways of prescribed antipsychotics, namely, *CYP2D6*, *CYP3A4*, *CYP3A5*, *CYP1A2*, *ABCB1*, and *ABCG2*. Treatment recommendations for drug–gene pairs were made according to available evidence-based pharmacogenetic recommendations from the Dutch Pharmacogenetics Working Group (DPWG) or Clinical Pharmacogenetics Implementation Consortium (CPIC).

**Results:**

Pharmacogenetic testing revealed a specific metabolic profile and pharmacokinetic phenotype of the patient, which in retrospect provided possible explanations for the observed ADRs. Based on the pharmacogenetic results, the choice of an effective and safe medication proved to be much easier. The psychotic symptoms disappeared after treatment, while the negative symptoms persisted to a lesser extent.

**Conclusion:**

With the case presented, we have shown that taking into account the pharmacogenetic characteristics of the patient can explain the response to antipsychotic treatment and associated side effects. In addition, pharmacogenetic testing enabled an informed choice of the most appropriate drug and optimal dose adjustment. This approach makes it possible to avoid or minimize potentially serious dose-related ADRs and treatment ineffectiveness. However, due to the complexity of psychopathology and the polypharmacy used in this field, it is of great importance to conduct further pharmacokinetic and pharmacogenetic studies to better assess gene–drug and gene–gene–drug interactions.

## Introduction

Antipsychotics are used to treat various psychiatric disorders, including schizophrenia, bipolar disorder, and major depression (1). Most antipsychotics are metabolized in the liver, primarily by cytochrome P450 (CYP) enzymes, particularly CYP2D6, CYP3A4, CYP1A2, and CYP2C19. These enzymes exhibit considerable genetic polymorphism, which leads to significant inter-individual differences in response to antipsychotic drugs (2). In particular, CYP2D6 plays an important role in the metabolism of approximately 40% of antipsychotics (3). Differences in enzyme activity can be attributed to genetic variability and lead to variations in drug or metabolite concentration and response to treatment. In a study conducted in Norwegian patients, the effects of CYP2D6 variations on exposure to the active substance of risperidone and aripiprazole were quantified. The results showed that the *CYP2D6* genotype has a significant impact on the metabolism of risperidone and aripiprazole. Specifically, intermediate metabolizers showed an approximately 1.4-fold increase and poor metabolizers a 1.6-fold increase in the active moiety of risperidone and aripiprazole compared to normal metabolizers (4). Given the narrow therapeutic window of risperidone and the relatively long half-life of aripiprazole, these patients are more likely to experience dose-related adverse drug reactions (ADRs) (5).

It is important to note that ADRs are among the top 10 causes of mortality and morbidity in industrialized countries (6). Approximately one in 10 adult emergency department visits in the United States from 2009 to 2011 were attributed to adverse effects of psychotropic drugs (7). In addition, treatment resistance to pharmacotherapy is relatively common in psychiatry. Approximately 30% of patients with schizophrenia and related disorders meet the criteria for a treatment-resistant disorder (8). This suggests that there are different subgroups of schizophrenia spectrum disorders defined by their response to therapy. This concept was originally introduced by Kane (9), who focused on patients who exhibit resistance to most antipsychotic medications. However, further studies have shown that pharmacokinetic factors play a crucial role in medication efficacy, especially in conjunction with poor adherence to treatment, which leads to subtherapeutic plasma levels in approximately one-third of cases (10). Poor adherence to psychopharmacotherapy is a common problem affecting approximately half of patients with severe psychiatric disorders. The main factors contributing to poor adherence include lack of insight, ineffectiveness of treatment, and the complexity of taking multiple medications (polypharmacy) (11). Although the exact impact of side effects on treatment adherence remains unclear, they significantly influence a psychiatrist’s decision-making process in medication selection (12).

In this case report, we discuss a therapeutic challenge we encountered in a 60-year-old female patient with psychosis. She suffered from severe extrapyramidal ADRs when she was treated with risperidone and fluphenazine. In addition, during treatment with aripiprazole, brexpiprazole, and olanzapine, she developed an incipient malignant neuroleptic syndrome with potentially life-threatening complications that required admission and treatment in the intensive care unit. After her physical condition had improved, her psychosis remained active, and there was an urgent need for treatment. However, the selection of an appropriate antipsychotic became extremely difficult after these complications. Ultimately, our dilemma was effectively resolved through the use of pharmacogenetic analysis.

## Case description

A 60-year-old woman was referred to a psychiatric outpatient clinic for the first time by her general practitioner (GP) in March 2019 due to observed psychotic symptoms. She had not received psychiatric treatment in the past, was a non-smoker, denied abusing psychoactive substances, and stated that she had no family history of psychiatric disorders. Delusions of persecution and auditory hallucinations were observed, and low-dose risperidone treatment was initiated, initially at 1 mg/day, and at a subsequent visit, the dose was increased to 2 mg/day. The results of a clinical–psychological examination showed a pattern consistent with chronic psychosis, while a CT scan of the head revealed no structural abnormalities, and only minimal atrophy of the frontal cortex was noted. She attended regular check-ups over a period of approximately 2 years. At subsequent visits, she reported that she was only partially compliant with the prescribed medication. She also described symptoms that were interpreted as possible extrapyramidal ADRs but not confirmed. For example, she expressed a feeling of stiffness by stating, “When I was on the medication, even sitting down on a chair became extremely difficult for me”.

In July 2022, she was voluntarily admitted to the psychiatric intensive care unit at the University Psychiatric Clinic Ljubljana. This decision was triggered by her own call to the emergency services, in which she expressed her concern that she was in danger. She exhibited delusions of persecution and auditory and possibly cenesthetic hallucinations, accompanied by disorganized behavior. Standard laboratory tests of blood and urine revealed no significant abnormalities. A clinical–psychological examination confirmed these observations; however, a more comprehensive assessment of cognitive function was not possible due to the severe formal thought disorder, suggesting an acute exacerbation in the context of a most likely long-standing psychosis. During a 4-week hospitalization, treatment with risperidone was gradually resumed, and the dosage was gradually escalated to 9 mg/day due to a lack of clinically evident improvement in terms of positive symptoms. Following an insufficient response despite the increased dose, the decision to augment the treatment was taken, and fluphenazine was initiated informed by the understanding that further escalation of the risperidone dose, without available pharmacogenetic data or plasma levels for risperidone and paliperidone, would be unreasonable due to the fact that higher doses are reportedly not superior in terms of efficacy (13). Subsequently, there was a remission of positive symptoms. On discharge, she was prescribed risperidone at a daily dose of 9 mg, divided into two doses, together with fluphenazine at a dose of 10 mg per day, also divided into two doses. At the time of discharge, there were no clinically recognizable extrapyramidal symptoms (EPSs) or akathisia.

After her discharge in August 2022, the patient was readmitted to our clinic in October 2022 after neighbors, who had heard her cries for help, initiated an emergency response that led to a forced entry into her apartment. There was a total of 51 days between her discharge and re-admission. She reported that she was unable to move or get out of bed for 2 days and was not even able to get food. The description suggested possible severe EPSs from the prescribed antipsychotic. She stated that she had been taking the medication regularly. A subsequent examination in the psychiatric emergency room revealed no signs of acute psychosis. The neurological examination revealed bradykinesia and rigidity with a positive cogwheel sign. Due to the rigidity noted, the dose of fluphenazine was reduced on admission and discontinued the next day. In addition, the dose of risperidone was reduced to 6 mg/day on admission and gradually reduced to 2 mg/day divided into two doses over the following 3 days, with biperiden being introduced simultaneously at a dose of 4 mg/day divided into two doses. On the second day of this treatment, respiratory distress occurred, characterized by tachypnea with a respiratory rate of 20 breaths/min, tachycardia, and no fever. Auscultation revealed diffuse crackles and bronchial breathing. Blood oxygen saturation dropped to 80%, and she was drowsy but continued to respond to direct verbal contact. She was urgently referred to an internal medicine emergency department, where laboratory tests revealed elevated inflammatory markers and an elevated white blood cell count. A chest X-ray showed infiltrates in the right lung, which led to the diagnosis of pneumonia. Empirical antibiotic therapy, consisting of amoxicillin with clavulanate and azithromycin, was initiated immediately. After 10 days of antibiotic treatment, her symptoms improved, she no longer required oxygen therapy, and a normalization of laboratory parameters was observed.

During this time, her mental state deteriorated, marked by a recurrence of acute psychosis characterized by considerable disorganization and delusions of persecution. Due to persistent EPSs at a low risperidone dose, aripiprazole was initially prescribed at a dosage of 5 mg/day and later increased to 10 mg/day, while risperidone was gradually discontinued. At this time, she was concomitantly receiving biperiden at a dosage of 6 mg/day for persistent bradykinesia, hypomimia, and rigidity, with pharyngeal and laryngeal muscle involvement observed, resulting in dysphagia and dysphonia. She also exhibited marked restlessness and an inability to keep still. Nursing staff documented increasing psychomotor agitation leading to insomnia, while she reported significant subjective complaints suggestive of possible iatrogenic akathisia, with a Barnes Akathisia Rating Scale of 9/9. Lorazepam was introduced at a dose of 1 mg three times daily and later 2 mg twice daily. Aripiprazole was replaced by brexpiprazole at a dose of 1 mg, which was chosen because of its lower incidence of akathisia (14). Subsequently, olanzapine was introduced at a dose of 5 mg to better manage the still-pronounced symptoms of acute psychosis while avoiding a possible exacerbation of EPSs.

Despite the change in antipsychotic therapy and the introduction of lorazepam as well as continued treatment with biperiden, both the akathisia and the pronounced rigidity escalated the next day. Subsequently, all antipsychotic medications were temporarily discontinued, as an incipient neuroleptic malignant syndrome was suspected, which was confirmed by the elevated creatine kinase and myoglobin levels detected. In addition, acute respiratory distress occurred again, accompanied by a drop in blood oxygen levels, fever, and elevated inflammatory markers. An infection with the H1N1 subtype of the influenza A virus was diagnosed. She was treated with oseltamivir and later empirically with amoxicillin with clavulanate, as secondary bacterial pneumonia was suspected, probably due to aspirations related to the persistent dysphagia. Her physical health continued to deteriorate, and she was transferred to a specialist infectious disease clinic for intensive care and continued treatment. A chest X-ray revealed infiltrates in the left basal and right hilobasal area and right middle lobe, while sputum analysis was positive for *Enterobacter cloacae* and *Staphylococcus aureus*. It is important to note that the chest X-ray exhibited right-sided lobar infiltrates in both cases of pneumonia, providing further credence to the notion of aspiration, considering the intricacies of the tracheobronchial anatomy.

**Figure 1:**
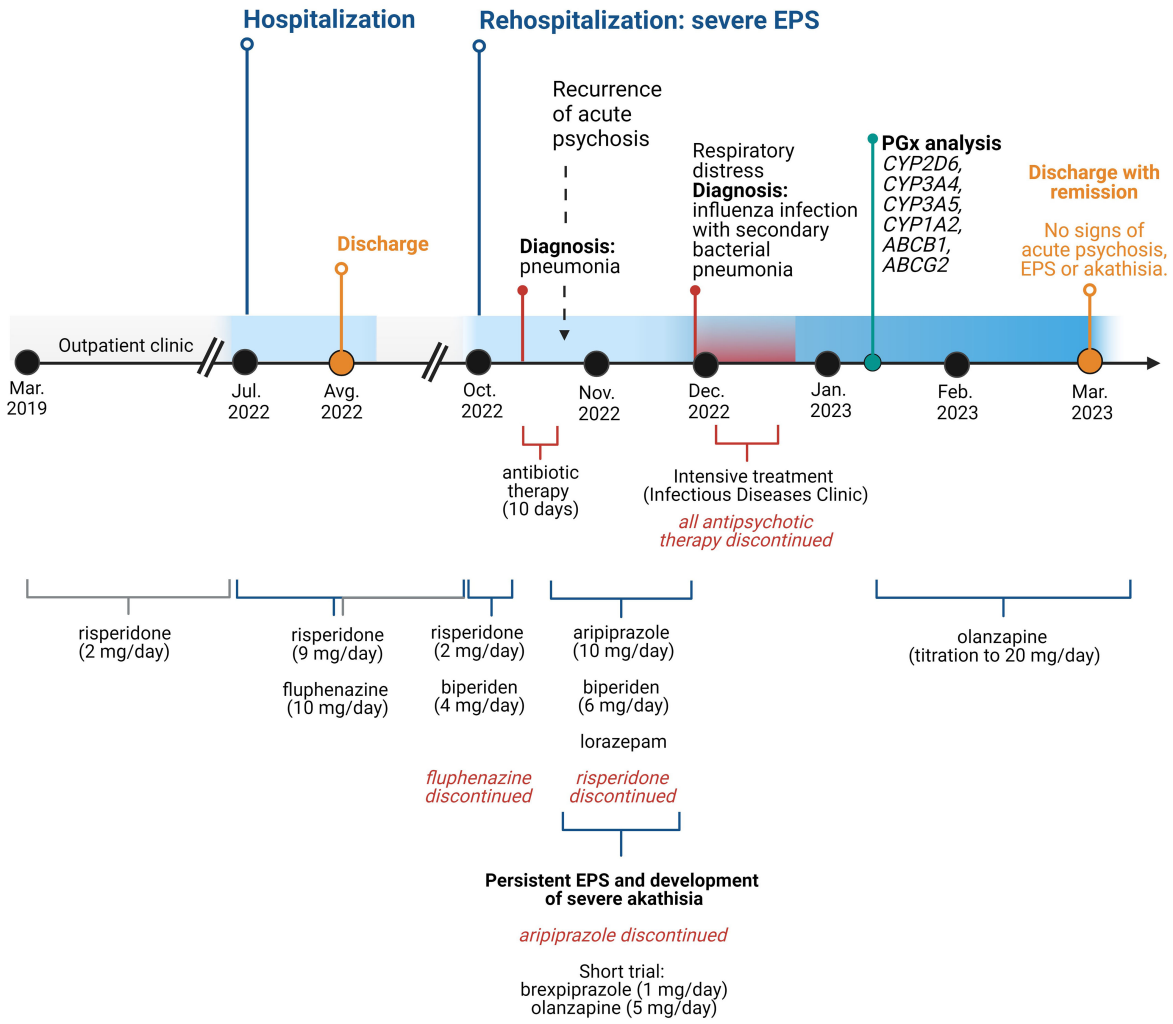
Time course of the treatment. EPS, extrapyramidal symptom. Created with BioRender.com.

After her physical condition had stabilized, psychiatric treatment was continued in our clinic. A dopamine transporter scintigraphy (DAT scan) revealed no evidence of presynaptic dopaminergic dysfunction, confirming the clinical suspicion that the observed extrapyramidal symptoms were indeed iatrogenic. The overall time course of treatment is summarized in **Figure 1**.

**Figure 2:**
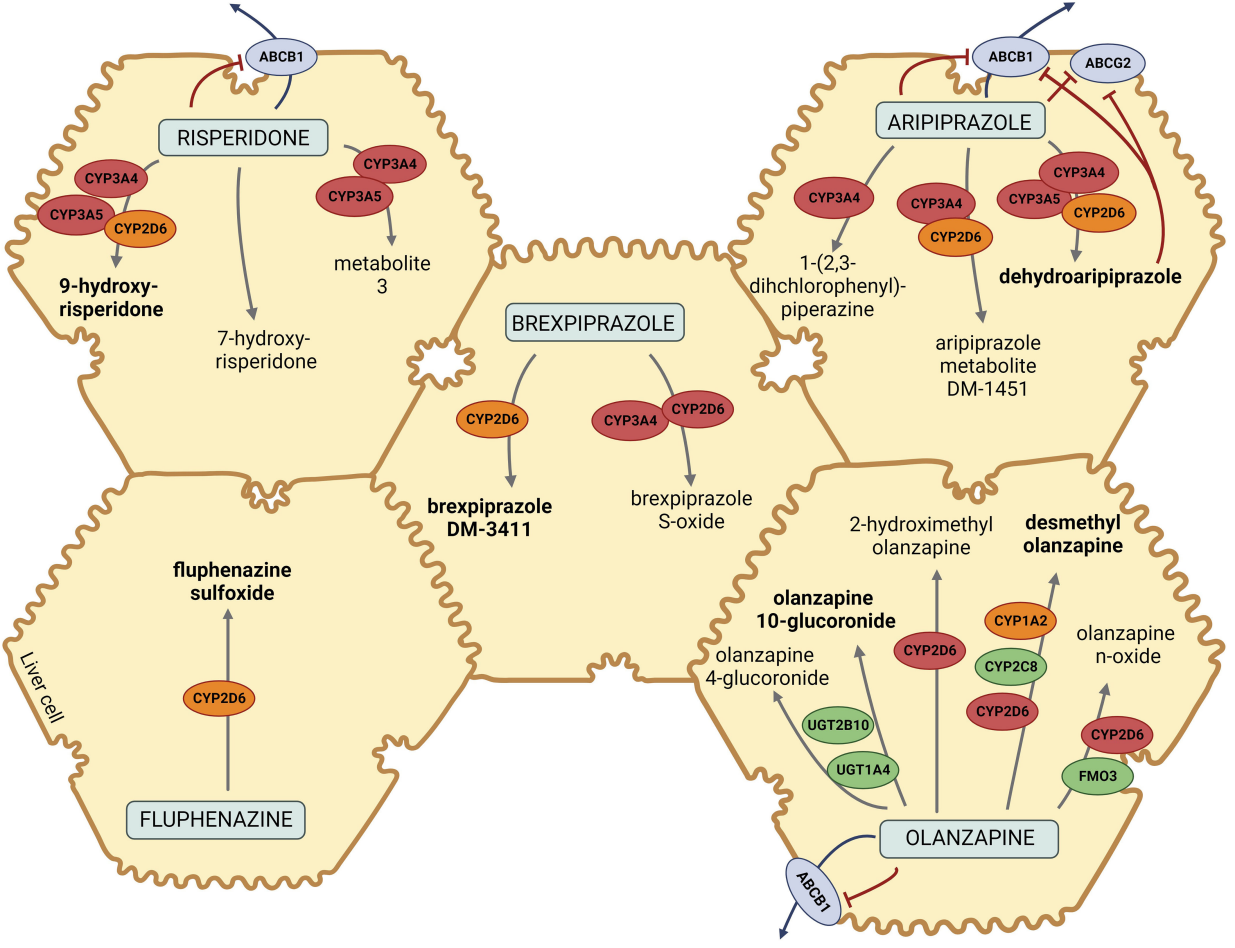
Pharmacokinetic pathways of risperidone, brexpiprazole, aripiprazole, fluphenazine, and olanzapine. Metabolites in bold are the major metabolites of designated drugs. CYP2D6, cytochrome P450 Family 2 Subfamily D Member 6; CYP3A4/5, cytochrome P450 Family 3 Subfamily A Member 4/5; CYP2C8, cytochrome P450 Family 2 Subfamily C Member 8; CYP1A2, cytochrome P450 Family 1 Subfamily A Member 2; ABCB1, ATP-binding cassette sub-family B member 1; ABCG2, ATP-binding cassette super-family G member 2; UGT1A4, Uridine 5′-diphospho-glucuronosyltransferase Family 1 Subfamily A Member 4; UGT2B10, Uridine 5′ diphospho-glucuronosyltransferase Family 2 Subfamily B Member 10; FMO3, flavin monooxygenase 3. The figure was created with BioRender.com and was inspired based on Whirl-Carrillo et al. (15) and Soria-Chacartegui et al. (16).

Given the complicated clinical presentation and increased susceptibility to the development of EPSs, we hypothesized that a specific pharmacokinetic profile could explain these ADRs. Based on the pharmacokinetic pathways of the majority of prescribed drugs causing ADRs (**Figure 2**), pharmacogenetic testing was performed by analyzing common functional variants in *CYP2D6*, *CYP3A4*, *CYP1A2*, *CYP3A5*, *ABCB1*, and *ABCG2*.

## Methods

For pharmacogenetic testing, the patient’s DNA was isolated from the cellular residue (leukocytes) from a peripheral blood sample collected in ethylenediaminetetraacetic acid (EDTA)-containing vacutainer tubes using the E.Z.N.A. The uniformity in an individual’s genetic makeup, regardless of the cell type—be it cells from peripheral blood or liver cells—ensures that genetic analysis conducted using peripheral blood is applicable for studying hepatic enzyme genes.

SQ Blood Kit II (Omega Bio-Tek, Norcross, GA, USA) was used according to the manufacturer’s instructions. The presence of *CYP2D6* gene deletion (*5) or duplication (*xN) was tested using long-range PCR (Long-Range PCR, Biotechrabbit GmbH, Berlin, Germany). Analysis of *ABCB1* 2677T>G/A polymorphisms was determined by using an allele-specific PCR performed in three separate amplification reactions with a set of allele-specific primers as described by Kurzawski et al. (17). The CYP2D6*5, xN and *ABCB1* 2677T/G/A amplicons were visualized after agarose gel electrophoresis. All other genetic variants (CYP1A2*1F; CYP3A4*1B, *22; CYP3A5*3, *6, *7; CYP2D6*3, *4, *6, *8, *9, *10, *14A/B, *17, *41; *ABCB1* 1236C>T, 3435C>T; and *ABCG2* 421C>A, 34G>A) were analyzed using commercial KASP SNP Genotyping Assay kits (LGC Group, Middlesex, UK), which enable the detection of products using fluorescence. All analyses were performed in duplicate in the presence of appropriate negative and positive controls for all genotypes and additional control samples.

## Results

As demonstrated in **Figure 2**, the majority of the prescribed atypical antipsychotics (risperidone, aripiprazole, and brexpiprazole) are predominantly metabolized via CYP2D6 and CYP3A4, while olanzapine is metabolized via CYP1A2 and, to a lesser extent, via CYP2D6. Fluphenazine, a typical antipsychotic, is also metabolized via CYP2D6. According to the literature and pharmacokinetic pathways, *CYP3A5*, *ABCB1*, and, *ABCG2* could also contribute to a patient’s treatment response (15). The results of genotyping of *CYP1A2*, *CYP3A4*, *CYP3A5*, *CYP2D6*, *ABCB1*, and *ABCG2* polymorphisms are presented in **Table 1**.

**Table 1:** Summary of the genotyping results and the patient’s phenotypes.

Gene	The analyzed polymorphisms	Patient’s genotype	Polymorphic allele’s effect	Patient’s phenotype
*CYP1A2*	*1F	*1F/*1F	Increased enzyme activity	UM (DPWG)
*CYP3A4*	*1B, *22	*1/*1	Normal function	NM
*CYP3A5*	*3, *6, *7	*3/*3 ^#^	Decreased enzyme activity	PM^#^
*CYP2D6*	*3, *4, *5, *6, *8, *9, *10, *14A/B, *17, *41, xN	*1/*41	Decreased enzyme activity	NM (DPWG, CPIC)
*ABCB1*	1236T>C, 2677T>G/A, 3435T>C	1236**CC**, 2677**GA**, 3435**CC**,	Haplotype C-G-C	–
*ABCG2*	421C>A, 34G>A	421CC, 34GG	Normal function	NM (DPWG)

NM, normal metabolizer; IM, intermediate metabolizer; RM, rapid metabolizer; UM, ultra-rapid metabolizer; DPWG, assigned by Dutch Pharmacogenetics Working Group; CPIC, assigned by the Clinical Pharmacogenetics Implementation Consortium.

^#^The most prevalent genotype/phenotype in EU population (15).

The genotyping results indicated the presence of genetic variations leading to altered drug metabolism. The presented patient is a carrier of one polymorphic CYP2D6*41 allele and is therefore considered a normal metabolizer (NM) for CYP2D6. However, CYP2D6*41 polymorphism may result in lower enzyme activity. The patient is also an ultra-rapid metabolizer (UM) for *CYP1A2* (homozygous for CYP1A2*1F) and could therefore be susceptible to decreased drug efficacy since this phenotype causes increased enzyme activity resulting in faster inactivation of active compounds. The patient is a carrier of two polymorphic CYP3A5*3 alleles with zero CYP3A5 activity and is therefore a poor metabolizer (PM). However, this phenotype is the most prevalent in the European population and is considered a “normal/expected” variation. None of the analyzed polymorphisms in *CYP3A4* (*1B and *22) were found. Analysis of the ATP-binding cassette (ABC) transporters confirmed the presence of *ABCB1* 1236T>C, 3435T>C, and 2677T>G/A, but not *ABCG2* 402 A>C and 34G>A polymorphisms.

## Discussion

In the case presented, our patient suffered from severe EPSs while being treated with several antipsychotics. This was aggravated by pneumonia, which probably developed as a result of aspiration due to EPS-related dysphagia. An incipient neuroleptic malignant syndrome was also suspected. We were able to explain the patient’s response to the prescribed antipsychotics to some extent by her pharmacogenetic profile.

Risperidone was the first antipsychotic prescribed to the patient. During her first treatment with risperidone, she reported symptoms that were retrospectively interpreted as EPSs. These were also observed later, at the time of her second hospitalization. In terms of efficacy, risperidone was not sufficiently effective even when the dose was increased to 9 mg/day, so fluphenazine had to be added to the treatment regimen. Risperidone has a lower potential to cause EPSs compared to typical antipsychotics. The major metabolic pathway of risperidone is 9-hydroxylation, with conversion to 9-hydroxyrisperidone (paliperidone) catalyzed by CYP2D6 and CYP3A4. This metabolite is equipotent to risperidone in terms of dopamine receptor affinity and therefore contributes to the overall therapeutic effect of risperidone (18). The relationship between risperidone dosage and the occurrence of EPSs is directly proportional; i.e., as the risperidone concentration increases, the likelihood of EPSs increases as well (19). The relatively high dose of risperidone, especially coupled with the addition of fluphenazine, in itself, could account for EPSs. Risperidone and fluphenazine can of course have additive effects due to their similar roles as D2 antagonists, increasing the risk of ADR-like tremors, rigidity, and anticholinergic symptoms (e.g., dry mouth and blurred vision). Other potential additive side effects like QT prolongation or sedation were not observed. While it is important to consider how these drugs affect each other’s metabolism, specific interactions between risperidone and fluphenazine are not well-established (20). However, in this specific case, the pharmacogenetic profile offers further insight into the mechanism of both inefficacy and the curiously delayed presentation of EPSs, as no EPSs were reported at discharge following her first hospitalization, but she later presented with severe EPSs.

There are currently no recommendations for dose adjustment of risperidone in the CYP2D6*1/*41 genotype (NM). However, the reduced enzyme activity suggests that drug–gene interactions may cause the occurrence of ADRs during risperidone treatment, although no clinically relevant associations have been demonstrated to date (21). It is known that plasma concentrations of 9-hydroxyrisperidone are reduced in carriers of CYP2D6*41, implying higher plasma concentrations of the parent compound leading to higher plasma concentrations of the parent drug (4, 21). Due to lower polarity, risperidone can cross the blood–brain barrier more effectively than 9-hydroxyrisperidone, which may explain the development of ADRs associated with the central nervous system and thus EPSs (22).

In addition, several studies have shown that the response to treatment with risperidone could also be explained by *ABCB1* polymorphisms. *ABCB1* encodes glycoprotein P (P-gp), which is involved in the absorption, distribution, and excretion of risperidone (23). Xing et al. reported that the 1236TT genotype is associated with a better response to risperidone treatment (24), while a study by Mi et al. (2016) showed that carriers of the C allele *ABCB1* 1236T>C had a significantly higher total EPSs incidence rate than TT carriers when treated with risperidone. They observed similar risperidone treatment outcomes for GT or GA carriers of *ABCB1* 2677T>G/A compared to other genotype carriers (25). In addition, they used a haplotype analysis to show that C-G-C carriers (3435T>C-2677T>G/A-1236T>C) were more likely to suffer from tremors than non-carriers. A lower response to long-acting intramuscular risperidone in 1236 CC, 3435 CC, or 2677GG carriers (2677T>G/A) was also reported by Ganoci et al., but they did not observe any effects of *ABCB1* polymorphisms on risperidone exposure (26). However, not all studies have confirmed these findings, so the results should be interpreted with caution. Nevertheless, both EPSs and a partial response to risperidone treatment could possibly be explained by considering the genotypes of the patient (CYP2D6*41 and *ABCB1* 1236CC, 2677GA, 3435CC with a C-G-C haplotype).

When speculating on the role of *CYP2D6* *41 in risperidone and aripiprazole treatment, it is important to note that the assignment of phenotypes is based on the assessment of the impact of a particular allele on metabolism. The current classification attributes only a minor effect of the CYP2D6*41 allele on the CYP2D6 metabolic activity (0.5). However, some studies have reported that CYP2D6*41 reduces the activity of the enzyme to a much greater extent as agreed and confirmed by the Clinical Pharmacogenetics Implementation Consortium (CPIC) and Dutch Pharmacogenetics Working Group (DPWG). For example, Jukić et al. (27–29) demonstrated that the activity score for the CYP2D6*41 allele is more likely close to 0.18, particularly when risperidone, aripiprazole, or venlafaxine are used (0.14, 0.26, and 0.21, respectively), and is not equal to 0.5 as assumed by the current classification. Similar results were observed in a study by Haslemo et al. (29), who found that the score for residual enzyme activity was only 9.5% (0.095) when venlafaxine was administered. This was also confirmed by Jin et al. (30), who demonstrated that the CYP2D6*41 allele has a significant effect on the metabolic activity of CYP2D6, regardless of the urinary, plasma, or salivary phenotyping method used, and has a similar or greater effect on CYP2D6 enzyme activity than CYP2D6*10. In our previous case report, we also observed that a patient with CYP2D6*1/*41 genotype responded unfavorably to treatment with risperidone, aripiprazole, and venlafaxine, although she was treated with the standard doses recommended for NM (31). In view of these data, the patient presented could be classified as IM and not NM. Regardless of the speculation, with a *CYP2D6* *1/*41 genotype defined as NM, treatment recommendations for risperidone, aripiprazole, or brexpiprazole do not apply to our patient. Furthermore, not all studies are conclusive regarding the role of *ABCB1* polymorphisms in risperidone/aripiprazole treatment. Therefore, more studies are needed to further investigate the role of these polymorphisms in the pharmacokinetics of antipsychotics. Finally, it is important to emphasize that other less common or rare variants of pharmacogenes could also play an important role in altered metabolism and response to treatment but were not analyzed in our patient.

In addition to risperidone, fluphenazine was added to the treatment regimen during the first hospitalization. We believe that there may have been a gradual increase in the concentration of risperidone and fluphenazine in the 2 months prior to the patient’s re-admission, which may have subsequently led to dose-related EPSs. These ADRs could also in part be due to decreased CYP2D6 activity and impaired metabolism of risperidone and fluphenazine. This could theoretically lead to a slower increase and delay in achieving the steady state of the active metabolite, paliperidone. Since both molecules possess nearly equivalent antipsychotic activity, their sum trough concentrations represent the active moiety accounting for both efficacy and ADRs. It is possible that both the initial inefficacy and subsequent delayed EPSs could in part be due to a delayed increase in paliperidone concentrations and consequently delayed increase in the active moiety.

However, it is important to note that the psychotic symptoms had improved after the combination therapy was prescribed and that the therapy was still effective at the time of her second admission, as at that time she did not present with psychotic symptoms but rather with severe extrapyramidal ADRs and psychotic symptoms only reappeared after the discontinuation of fluphenazine and dose reduction of risperidone. At this point, the possibility of dopamine supersensitivity psychosis was carefully considered. However, the improvement rather than exacerbation of extrapyramidal ADRs with the reduction in D2 antagonist dosage, in contrast to the typical trajectory observed with tardive phenomena, suggests that an underlying clinically relevant postsynaptic supersensitivity may be less probable (32).

On her second admission, a new treatment regimen was introduced due to the severe EPSs. She was given aripiprazole but developed severe akathisia at a dose of 10 mg, which led to additional symptomatic therapy with lorazepam. Due to persistent ADRs, aripiprazole was replaced by brexpiprazole, and later, olanzapine was introduced. Soon, all antipsychotic medications were temporarily discontinued due to the suspicion of an incipient neuroleptic malignant syndrome and later pneumonia. It is therefore important to note that while the patient was receiving antibiotics, the antipsychotic treatment was paused. This minimizes the potential interactions between the antibiotics and the enzymes that metabolize antipsychotics. It is also important to consider that the inflammatory mediators associated with pneumonia can affect the activity of CYP enzymes, thereby impacting the adverse drug reactions of antipsychotics. Nonetheless, in this specific case, the side effects occurred prior to the development of pneumonia.

The patient’s pharmacogenetic properties may partly explain the response to aripiprazole, brexpiprazole, and olanzapine. All are atypical antipsychotics, of which aripiprazole and brexpiprazole are metabolized primarily via CYP2D6 and CYP3A4, whereas olanzapine is metabolized via CYP1A2 and to a lesser extent via CYP2D6 (21, 33). There are evidence-based pharmacogenetic recommendations for the *CYP2D6*–aripiprazole and *CYP2D6*–brexpiprazole gene–drug pairs, but only for the PMs. These recommendations state that patients with a *CYP2D6* PM phenotype should not be administered more than 10 mg/day or 300 mg/month (68%–75% of the normal maximum dose of aripiprazole) or, in the case of brexpiprazole, only half the normal dose (21). There are no pharmacogenetic recommendations available for the olanzapine–*CYP2D6* drug–gene pair. However, olanzapine metabolism may also be impaired to some extent in carriers of the CYP2D6*41 allele.

Both aripiprazole and its active metabolite dehydroaripiprazole are substrates of P-gp (34). According to the literature, the relationship between patients’ *ABCB1* genotypes and the occurrence of ADRs cannot be confirmed. Belmonte et al. reported a significantly lower clearance of aripiprazole, together with lower AUC_0−t_ and C_max_ of dehydro-aripiprazole in subjects with *ABCB1* 1236TT genotype compared to 1236CC genotype carriers. They also showed that although the *ABCB1* SNPs studied had no significant effect on the AUC_0−t_ or C_max_ of aripiprazole, the rs1045642 T allele, the rs2235048 C allele, and the rs1045642–rs2235048–rs1128503–rs2032582 T-C-T-A haplotype were significantly associated with decreased sympathetic activity in these patients (35). Both findings suggest that patients with the 1236TT genotype may be more sensitive to aripiprazole and consequently more susceptible to developing ADRs. In contrast, in a study by Ivaschenko et al., an association between the *ABCB1* 3435 CC genotype and more pronounced akathisia was observed in adolescents treated with antipsychotics for acute psychotic episodes (36) Again, not all studies agreed or were able to demonstrate the role of ABCB1 in the pharmacokinetics of aripiprazole (37), and these results must also be interpreted with caution. In this particular case, the described akathisia could theoretically be attributed not only to the direct consequences of aripiprazole’s intrinsic activity but also to the withdrawal akathisia resulting from the reduction in risperidone dosage. The subsequent substitution of risperidone with aripiprazole in the receptor–antagonist complex may induce a net effect akin to the reduction of the antagonist dosage, offering an alternative perspective on the etiology of the observed akathisia.

In contrast, studies on olanzapine and *ABCB1* may support the observed response to some extent. ADRs were suspected with olanzapine, but it was later found that higher doses were required. In terms of olanzapine efficacy, Bozina et al. (38) reported that the T allele and TT genotype of G2677T (rs2032582) are associated with better response to olanzapine treatment in female schizophrenic patients. A better clinical response, as measured by the PANSS, was also observed by Yan et al. in the T allele of *ABCB1* C3435T (rs1045642) (39). In addition, both variants together with the C1236T T allele form the *ABCB1* TTT haplotype, which is associated with higher olanzapine concentrations. Patients with this haplotype may require lower doses of olanzapine than carriers of the non-TTT haplotype to achieve the desired response (40). Since our patient is a carrier of alleles 3435 C and 2677 G, a lower efficacy of olanzapine could be expected as a consequence of the *ABCB1* variations. However, this does not explain ADRs induced after olanzapine initiation in our patient. As not all studies were significant or concordant, these results should also be taken with caution. Since CYP1A2 is the main metabolic enzyme involved in olanzapine metabolism and our patient was homozygous for the CYP1A2*1F polymorphism, it is more likely that the response to olanzapine treatment or the need for higher doses is related to this polymorphism, which is located in the promoter region of *CYP1A2* gene and increases the inducibility of the gene and thus its expression (41). This leads to increased olanzapine metabolism and a poorer response to treatment.

Based on the pharmacogenetic results, it was decided to reintroduce olanzapine, with careful titration to a daily dose of 20 mg. Over the following weeks, the signs of psychotic disorganization gradually diminished. Persecutory ideation no longer occurred, but a notable degree of negative symptomatology persisted, characterized by avolition, diminished interest in social interactions, and blunted affect. No EPSs or akathisia was reported, and no additional treatment with biperiden or lorazepam was required. Following this improvement, the patient was discharged, and treatment continued in our outpatient service.

## Conclusions

The use of a pharmacogenetic profile in the context of personalized psychopharmacotherapy simplifies the process of selecting the right drug and the optimal dosage. The conventional approach to psychopharmacotherapy, characterized by numerous trials and errors, is not only time-consuming but often leads to polypharmacy, medication switching, and the development of ADRs.

As this case report shows, the occurrence of ADRs with the potential for life-threatening complications could be attributed to pharmacogenetic factors. The case report demonstrates the importance of pharmacogenetic testing as a cornerstone of precision psychiatry to improve treatment efficacy, prevent potential ADRs, and promote overall patient wellbeing.

## Data availability statement

The data presented in the case report are available upon reasonable request. Further inquiries may be directed to the corresponding authors.

## Ethics statement

Ethical approval was not required for the case report involving humans in accordance with the local legislation and institutional requirements. Written informed consent was obtained from the individual for the publication of any potentially identifiable images or data included in this article.

## Author contributions

LK: Data curation, Formal analysis, Investigation, Methodology, Validation, Visualization, Writing – original draft, Writing – review & editing, Project administration. TB: Formal analysis, Investigation, Methodology, Software, Visualization, Writing – original draft, Writing – review & editing, Validation. VD: Formal analysis, Methodology, Writing – original draft, Writing – review & editing, Supervision, Validation, Conceptualization. SR: Methodology, Writing – original draft, Writing – review & editing, Formal analysis, Software, Visualization. JB: Methodology, Software, Supervision, Validation, Writing – original draft, Writing – review & editing. MP: Conceptualization, Data curation, Investigation, Supervision, Validation, Writing – original draft, Writing – review & editing, Formal analysis, Funding acquisition, Methodology, Project administration, Resources.

## References

[1] Lally J MacCabe JH . Antipsychotic medication in schizophrenia: a review. Br Med Bull. (2015) 114:169–79. doi: 10.1093/bmb/ldv017 25957394

[2] Ravyn D Ravyn V Lowney R Nasrallah HA . CYP450 Pharmacogenetic treatment strategies for antipsychotics: A review of the evidence. Schizophr Res. (2013) 149:1–14. doi: 10.1016/j.schres.2013.06.035 23870808

[3] Walden LM Brandl EJ Tiwari AK Cheema S Freeman N Braganza N . Genetic testing for CYP2D6 and CYP2C19 suggests improved outcome for antidepressant and antipsychotic medication. Psychiatry Res. (2019) 279:111–5. doi: 10.1016/j.psychres.2018.02.055 29699889

[4] Jukic MM Smith RL Haslemo T Molden E Ingelman-Sundberg M . Effect of CYP2D6 genotype on exposure and efficacy of risperidone and aripiprazole: a retrospective, cohort study. Lancet Psychiatry. (2019) 6:418–26. doi: 10.1016/S2215-0366(19)30088-4 31000417

[5] Van Westrhenen R Aitchison KJ Ingelman-Sundberg M Jukić MM . Pharmacogenomics of antidepressant and antipsychotic treatment: how far have we got and where are we going? Front Psychiatry. (2020) 11:94. doi: 10.3389/fpsyt.2020.00094 32226396 PMC7080976

[6] Cacabelos R Naidoo V Corzo L Cacabelos N Carril JC . Genophenotypic factors and pharmacogenomics in adverse drug reactions. Int J Mol Sci. (2021) 22:13302. doi: 10.3390/ijms222413302 34948113 PMC8704264

[7] Hampton LM Daubresse M Chang HY Alexander GC Budnitz DS . Emergency department visits by adults for psychiatric medication adverse events. JAMA Psychiatry. (2014) 71:1006. doi: 10.1001/jamapsychiatry.2014.436 25006837 PMC4703317

[8] Howes OD McCutcheon R Agid O De Bartolomeis A Van Beveren NJM Birnbaum ML . Treatment-resistant schizophrenia: treatment response and resistance in psychosis (TRRIP) working group consensus guidelines on diagnosis and terminology. Am J Psychiatry. (2017) 174:216–29. doi: 10.1176/appi.ajp.2016.16050503 PMC6231547 27919182

[9] Kane J . Clozapine for the treatment-resistant schizophrenic: A double-blind comparison with chlorpromazine. Arch Gen Psychiatry. (1988) 45:789. doi: 10.1001/archpsyc.1988.01800330013001 3046553

[10] McCutcheon RA Pillinger T Mizuno Y Montgomery A Pandian H Vano L . The efficacy and heterogeneity of antipsychotic response in schizophrenia: A meta-analysis. Mol Psychiatry. (2021) 26:1310–20. doi: 10.1038/s41380-019-0502-5 PMC7610422 31471576

[11] Semahegn A Torpey K Manu A Assefa N Tesfaye G Ankomah A . Psychotropic medication non-adherence and its associated factors among patients with major psychiatric disorders: a systematic review and meta-analysis. Syst Rev. (2020) 9:17. doi: 10.1186/s13643-020-1274-3 31948489 PMC6966860

[12] Roose SP . Compliance: the impact of adverse events and tolerability on the physician’s treatment decisions. Eur Neuropsychopharmacol. (2003) 13 (13):85–92. doi: 10.1016/S0924-977X(03)00097-X 14550581

[13] Möller HJ . Risperidone: a review. Expert Opin Pharmacother. (2005) 6:803–18. doi: 10.1517/14656566.6.5.803 15934906

[14] Huhn M Nikolakopoulou A Schneider-Thoma J Krause M Samara M Peter N . Comparative efficacy and tolerability of 32 oral antipsychotics for the acute treatment of adults with multi-episode schizophrenia: a systematic review and network meta-analysis. Lancet. (2019) 394:939–51. doi: 10.1016/S0140-6736(19)31135-3 PMC6891890 31303314

[15] Whirl-Carrillo M Huddart R Gong L Sangkuhl K Thorn CF Whaley R . An evidence-based framework for evaluating pharmacogenomics knowledge for personalized medicine. Clin Pharmacol Ther. (2021) 110:563–72. doi: 10.1002/cpt.2350 PMC8457105 34216021

[16] Soria-Chacartegui P Villapalos-García G Zubiaur P Abad-Santos F Koller D . Genetic polymorphisms associated with the pharmacokinetics, pharmacodynamics and adverse effects of olanzapine, aripiprazole and risperidone. Front Pharmacol. (2021) 12:711940. doi: 10.3389/fphar.2021.711940 34335273 PMC8316766

[17] Kurzawski M Pawlik A Górnik W Droździk M . Frequency of common MDR1 gene variants in a Polish population. Pharmacol Rep PR. (2006) 58:35–40. 16531628

[18] Mannens G Huang ML Meuldermans W Hendrickx J Woestenborghs R Heykants J . Absorption, metabolism, and excretion of risperidone in humans. Drug Metab Dispos Biol Fate Chem. (1993) 21:1134–41. 7507814

[19] Schotte A Janssen PFM Gommeren W Luyten WHML Van Gompel P Lesage AS . Risperidone compared with new and reference antipsychotic drugs: in *vitro* and in *vivo* receptor binding. Psychopharmacol (Berl). (1996) 124:57–73. doi: 10.1007/BF02245606 8935801

[20] Gallego JA Nielsen J De Hert M Kane JM Correll CU . Safety and tolerability of antipsychotic polypharmacy. Expert Opin Drug Saf. (2012) 11:527–42. doi: 10.1517/14740338.2012.683523 PMC3384511 22563628

[21] Beunk L Nijenhuis M Soree B de Boer-Veger NJ Buunk AM Guchelaar HJ . Dutch Pharmacogenetics Working Group (DPWG) guideline for the gene-drug interaction between CYP2D6, CYP3A4 and CYP1A2 and antipsychotics. Eur J Hum Genet EJHG. (2023). doi: 10.1038/s41431-023-01347-3 PMC10923774 37002327

[22] Calarge CA Miller DD . Predictors of risperidone and 9-hydroxyrisperidone serum concentration in children and adolescents. J Child Adolesc Psychopharmacol. (2011) 21:163–9. doi: 10.1089/cap.2010.0038 PMC3080754 21486167

[23] Ejsing TB Pedersen AD Linnet K . P-glycoprotein interaction with risperidone and 9-OH-risperidone studied*in vitro*, in knock-out mice and in drug-drug interaction experiments. Hum Psychopharmacol Clin Exp. (2005) 20:493–500. doi: 10.1002/hup.720 16118767

[24] Xing Q Gao R Li H Feng G Xu M Duan S . Polymorphisms of the ABCB1 gene are associated with the therapeutic response to risperidone in Chinese schizophrenia patients. Pharmacogenomics. (2006) 7:987–93. doi: 10.2217/14622416.7.7.987 17054409

[25] Mi W Liu F Liu Y Du B Xiao W Li L . Association of ABCB1 gene polymorphisms with efficacy and adverse reaction to risperidone or paliperidone in han chinese schizophrenic patients. Neurosci Bull. (2016) 32:547–9. doi: 10.1007/s12264-016-0050-9 PMC5563830 27456824

[26] Ganoci L Trkulja V Živković M Božina T Šagud M Lovrić M . ABCB1, ABCG2 and CYP2D6 polymorphism effects on disposition and response to long-acting risperidone. Prog Neuropsychopharmacol Biol Psychiatry. (2021) 104:110042. doi: 10.1016/j.pnpbp.2020.110042 32682874

[27] Jukić MM Smith RL Molden E Ingelman-Sundberg M . Evaluation of the CYP2D6 haplotype activity scores based on metabolic ratios of 4,700 patients treated with three different CYP2D6 substrates. Clin Pharmacol Ther. (2021) 110:750–8. doi: 10.1002/cpt.2246 33792048

[28] Molden E Jukić MM . CYP2D6 reduced function variants and genotype/phenotype translations of CYP2D6 intermediate metabolizers: implications for personalized drug dosing in psychiatry. Front Pharmacol. (2021) 12:650750. doi: 10.3389/fphar.2021.650750 33967790 PMC8100508

[29] Haslemo T Eliasson E Jukić MM Ingelman-Sundberg M Molden E . Significantly lower CYP2D6 metabolism measured as the O/N-desmethylvenlafaxine metabolic ratio in carriers of CYP2D6*41 versus CYP2D6*9 or CYP2D6*10: a study on therapeutic drug monitoring data from 1003 genotyped Scandinavian patients. Br J Clin Pharmacol. (2019) 85:194–201. doi: 10.1111/bcp.13788 30312494 PMC6303206

[30] Jin Y Zhang S Hu P Zheng X Guan X Chen R . The impact of CYP2D6*41 on CYP2D6 enzyme activity using phenotyping methods in urine, plasma, and saliva. Front Pharmacol. (2022) 13:940510. doi: 10.3389/fphar.2022.940510 36110554 PMC9468644

[31] Pjevac M Redenšek Trampuž S Blagus T Dolžan V Bon J . Case report: application of pharmacogenetics in the personalized treatment of an elderly patient with a major depressive episode. Front Psychiatry. (2023) 14:1250253. doi: 10.3389/fpsyt.2023.1250253 37608991 PMC10440381

[32] Chouinard G Samaha AN Chouinard VA Peretti CS Kanahara N Takase M . Antipsychotic-induced dopamine supersensitivity psychosis: pharmacology, criteria, and therapy. Psychother Psychosom. (2017) 86:189–219. doi: 10.1159/000477313 28647739

[33] Bousman CA Stevenson JM Ramsey LB Sangkuhl K Hicks JK Strawn JR . Clinical pharmacogenetics implementation consortium (CPIC) guideline for CYP2D6, CYP2C19, CYP2B6, SLC6A4, and HTR2A genotypes and serotonin reuptake inhibitor antidepressants. Clin Pharmacol Ther. (2023) 114:51–68. doi: 10.1002/cpt.2903 37032427 PMC10564324

[34] Holthoewer D Kirschbaum KM Frisch J Hiemke C Schmitt U . Pharmacodynamic effects of aripiprazole and ziprasidone with respect to p-glycoprotein substrate properties. Pharmacopsychiatry. (2013) 46:175–80. doi: 10.1055/s-0033-1347176 23737243

[35] Belmonte C Ochoa D Román M Saiz-Rodríguez M Wojnicz A Gómez-Sánchez CI . Influence of * CYP 2D6* , * CYP 3A4* , * CYP 3A5* and * ABCB 1* polymorphisms on pharmacokinetics and safety of aripiprazole in healthy volunteers. Basic Clin Pharmacol Toxicol. (2018) 122:596–605. doi: 10.1111/bcpt.12960 29325225

[36] Ivashchenko DV Yudelevich DA Buromskaya NI Shimanov PV Deitch RV Akmalova KA . CYP2D6 phenotype and ABCB1 haplotypes are associated with antipsychotic safety in adolescents experiencing acute psychotic episodes. Drug Metab Pers Ther. (2022) 37(1):47–53. doi: 10.1515/dmpt-2021-0124 35385893

[37] Suzuki T Mihara K Nakamura A Kagawa S Nagai G Nemoto K . Effects of genetic polymorphisms of CYP2D6, CYP3A5, and ABCB1 on the steady-state plasma concentrations of aripiprazole and its active metabolite, dehydroaripiprazole, in Japanese patients with schizophrenia. Ther Drug Monit. (2014) 36:651–5. doi: 10.1097/FTD.0000000000000070 24682161

[38] Bozina N Kuzman MR Medved V Jovanovic N Sertic J Hotujac L . Associations between MDR1 gene polymorphisms and schizophrenia and therapeutic response to olanzapine in female schizophrenic patients. J Psychiatr Res. (2008) 42:89–97. doi: 10.1016/j.jpsychires.2006.10.002 17113599

[39] Yan P Song M Gao B Wang S Wang S Li J . Association of the genetic polymorphisms of metabolizing enzymes, transporters, target receptors and their interactions with treatment response to olanzapine in chinese han schizophrenia patients. Psychiatry Res. (2020) 293:113470. doi: 10.1016/j.psychres.2020.113470 32992097

[40] Zubiaur P Soria-Chacartegui P Villapalos-García G Gordillo-Perdomo JJ Abad-Santos F . The pharmacogenetics of treatment with olanzapine. Pharmacogenomics. (2021) 22:939–58. doi: 10.2217/pgs-2021-0051 34528455

[41] Fekete F Menus Á Tóth K Kiss ÁF Minus A Sirok D . CYP1A2 expression rather than genotype is associated with olanzapine concentration in psychiatric patients. Sci Rep. (2023) 13:18507. doi: 10.1038/s41598-023-45752-6 37898643 PMC10613299

